# Near-future ocean acidification does not alter the lipid content and fatty acid composition of adult Antarctic krill

**DOI:** 10.1038/s41598-019-48665-5

**Published:** 2019-08-26

**Authors:** Jessica A. Ericson, Nicole Hellessey, So Kawaguchi, Peter D. Nichols, Stephen Nicol, Nils Hoem, Patti Virtue

**Affiliations:** 1 Institute for Marine and Antarctic Studies, University of Tasmania, 20 Castray Esplanade, Battery Point, Tasmania 7004 Australia; 2 Antarctic Climate & Ecosystems Cooperative Research Centre, 20 Castray Esplanade, Battery Point, Tasmania 7004 Australia; 3 CSIRO Oceans and Atmosphere, Castray Esplanade, Battery Point, Tasmania 7004 Australia; 4 Cawthron Institute, 98 Halifax Street East, The Wood, Nelson, 7010 New Zealand; 5 Australian Antarctic Division, 203 Channel Highway, Kingston, Tasmania 7050 Australia; 6 Aker Biomarine, Oksenøyveien 10, P.O. Box 496, NO-1327, Lysaker, Norway

**Keywords:** Fatty acids, Animal physiology

## Abstract

*Euphausia superba* (Antarctic krill) is a keystone species in the Southern Ocean, but little is known about how it will respond to climate change. Ocean acidification, caused by sequestration of carbon dioxide into ocean surface waters (*p*CO_2_), alters the lipid biochemistry of some organisms. This can have cascading effects up the food chain. In a year-long laboratory experiment adult krill were exposed to ambient seawater *p*CO_2_ levels (400 μatm), elevated *p*CO_2_ levels mimicking near-future ocean acidification (1000, 1500 and 2000 μatm) and an extreme *p*CO_2_ level (4000 μatm). Total lipid mass (mg g^−1^ DM) of krill was unaffected by near-future *p*CO_2_. Fatty acid composition (%) and fatty acid ratios associated with immune responses and cell membrane fluidity were also unaffected by near-future *p*CO_2_, apart from an increase in 18:3n-3/18:2n-6 ratios in krill in 1500 μatm *p*CO_2_ in winter and spring_._ Extreme *p*CO_2_ had no effect on krill lipid biochemistry during summer. During winter and spring, krill in extreme *p*CO_2_ had elevated levels of 18:2n-6 (up to 1.2% increase), 20:4n-6 (up to 0.8% increase), lower 18:3n-3/18:2n-6 and 20:5n-3/20:4n-6 ratios, and showed evidence of increased membrane fluidity (up to three-fold increase in phospholipid/sterol ratios). These results indicate that the lipid biochemistry of adult krill is robust to near-future ocean acidification.

## Introduction

*Euphausia superba* (Antarctic krill, hereafter ‘krill’) is a highly abundant keystone species in the Southern Ocean food web^1^. Their large biomass and dense swarms make them the primary food source for a range of Antarctic mega-fauna (whales, seals and penguins), fish, squid and seabirds. Krill are lipid-rich and contain high concentrations of nutritious long-chain (≥C_20_) polyunsaturated fatty acids (LC-PUFA), particularly eicosapentaenoic acid (20:5n-3) and docosahexaenoic acid (22:6n-3)^2,3^.

Krill are likely to be affected by anthropogenic climate change as ocean warming, sea level rise, sea ice melt and biological invasions increase^4^. Ocean acidification, caused by sequestration of atmospheric CO_2_ into ocean surface waters, may be particularly severe in polar marine ecosystems^5^. As atmospheric CO_2_ emissions rise, approximately 30% of these emissions are absorbed into seawater at the air/ocean interface^6^. This increase in seawater *p*CO_2_ alters the chemical equilibrium of seawater, causing a decrease in seawater pH^7^. Average ocean pH has already decreased by 0.1 units since the industrial revolution, and is predicted to decrease by another 0.33 units by the year 2300 if CO_2_ emissions are not mediated^8^.

Understanding how species may respond to ocean acidification can be assisted by long-term laboratory experiments which expose organisms to predicted future levels of seawater *p*CO_2_. Investigating how animals regulate their physiology in response to environmental changes can be examined through lipid biochemistry. Lipids and their associated fatty acids have a diverse range of roles in the metabolism; they are indicators of an organism’s diet and condition^9^, and can be used to detect biochemical shifts in response to stress^10,11^. Triacylglycerol (TAG) lipid classes are used for fat storage, while phospholipids (PL) and sterols (ST) make up the structure of cell membranes^12^. Long chain polyunsaturated fatty acids (LC-PUFA) such as 20:5n-3 (eicosapentaenoic acid) and 22:6n-3 (docosahexaenoic acid) are abundant anti-inflammatory fatty acids in PL membranes and conserved for reproductive processes^13,14^. Arachidonic acid (20:4n-6) is a less abundant but equally important LC-PUFA, being the primary precursor of eicosanoids, which regulate the immune system, reproductive processes, and ion flux^15^. Elevated ratios of n-6/n-3 PUFA in organisms are indicators of inflammation and physiological stress^16,17^.

Organisms can also alter their cell membrane fatty acid structure in response to environmental stressors^18^; a process termed ‘homeoviscous adaptation’. The effects of temperature on homeoviscous adaptation are well known^18^, but recent studies find that the mechanisms of homeoviscous adaptation can also be applied to other stressors such as pH. Bacteria alter their cell membrane fatty acid saturation and chain length as the pH level (acidity) of their environment changes, which may alter the permeability of the cell membrane and control proton influx^19–21^. The effects of decreasing seawater pH (via ocean acidification), and temperature on homeoviscous adaptation have recently been studied in marine sponges, by measuring ratios of polyunsaturated/saturated fatty acids (PUFA/SFA), ratios of PL/ST, and mean carbon chain length (MCL) in these organisms^11^. The kinked formation of PUFA increases membrane fluidity, while the absence of double bonds in densely packed SFA increases membrane stability^22^. Sterols can also be incorporated into cell membranes and packed between PUFA to increase membrane thickness^18^.

Increased *p*CO_2_, or a combination of increased *p*CO_2_ and temperature, have a range of effects on the lipid biochemistry of marine organisms: phytoplankton^23–26^ sponges^11^, fish^27,28^, crustaceans^29,30^, echinoderms^31,32^, corals^33^ and molluscs^34^.

Ocean acidification may result in a transition from high- to low-lipid phytoplankton and zooplankton species, and this could affect the fitness of higher predators^13,35^, promoting destabilization of the ecosystem.

Krill lipids have been widely studied due to the importance of krill in the food web^3,36–38^, and commercial interest from the Antarctic krill fishery^39,40^. A recent study showed that adult Antarctic krill are only affected by severe levels of *p*CO_2_^41^, but there is no published information on changes in krill lipid contents and fatty acid composition with elevated *p*CO_2_. It is essential to understand whether krill lipid composition will be affected by climate change because the Southern Ocean ecosystem is largely fuelled by lipid energy derived from krill.

Our study aimed to investigate the resilience of adult krill by observing the effects of elevated *p*CO_2_ on detailed aspects of krill lipid biochemistry. We reared krill for one year (January–December 2016) in ambient *p*CO_2_ levels (400 µatm *p*CO_2_), those predicted for the near-future (within the next 100–300 years; 1000, 1500 and 2000 µatm *p*CO_2_), and an extreme level of 4000 µatm *p*CO_2._ We analysed krill samples to observe whether the total lipid and fatty acid composition of krill changed with *p*CO_2_ over this long-term experiment. We also investigated whether lipid indicators of (a) homeoviscous adaptation (PUFA/SFA ratios, PL/ST ratios, and MCL) and (b) immune responses (n-3/n-6; 22:6n-3/20:4n-6 ratios and 18:3n-3/18:2n-6 ratios) in krill were altered by seawater *p*CO_2_.

## Results

### Effect of *p*CO_2_ on total lipid and phospholipid/sterol ratios in krill

**Figure 1:**
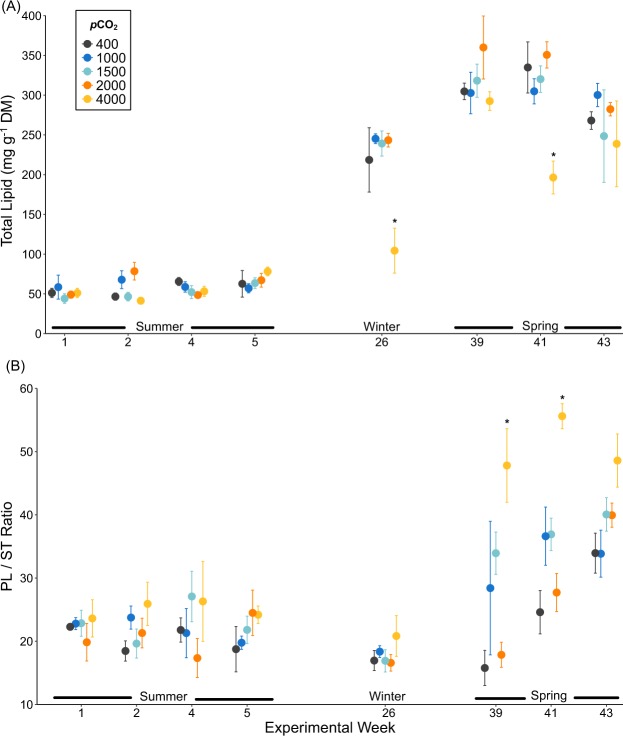
**(A)** Total lipid (mg g^−1^ dry mass; mean ± SE) and **(B)** phospholipid/sterol ratio (mean ± SE) of *Euphausia superba* in weeks 1 (January), 2 (February), 4 (February), 5 (February), 26 (July), 39 (October), 41 (November) and 43 (November) of the one-year ocean acidification experiment. For each *p*CO_2_ treatment and week n = 3–7. Statistically significant differences to ambient seawater (400 µatm *p*CO_2_) are highlighted with an asterisk (*p* < 0.05).

Quantities of total lipid in krill in weeks 1–5 did not differ between *p*CO_2_ treatments or weeks (*p*CO_2_; *p* = 0.577, week; *p* = 0.097; *p*CO_2_*week; *p* = 0.165). During these first five weeks of the experiment, average quantities of total lipid in krill (Fig. 1A) were 57.4 ± 19.8 mg/g dry mass (DM; mean ± SD). During weeks 26–43 (Fig. 1A), there was a fourfold increase in average total lipid in krill to 273.8 ± 75.4 mg/g DM (mean ± SD), and the effect of *p*CO_2_ on total lipid differed between weeks (Two Way ANOVA; *p*CO_2_*week, *p* = 0.052).

Krill in 4000 µatm *p*CO_2_ seawater had lower quantities of total lipid than krill in all other *p*CO_2_ treatments during week 26 (Tukey *p* < 0.003). They also had lower total lipid than krill in 400 and 2000 µatm *p*CO_2_ during week 41 (Tukey *p* < 0.046). During weeks 39 and 43, the quantities of total lipid in krill did not differ between *p*CO_2_ treatments (*p* > 0.930).

Ratios of PL/ST in krill (Fig. 1B) did not differ between *p*CO_2_ treatments during weeks 1–5 (Two Way ANOVA *p*CO_2_*week, *p* = 0.533). Krill in 4000 µatm *p*CO_2_ seawater had higher average PL/ST ratios than krill in 400 µatm *p*CO_2_ seawater during weeks 26–43, but these were significantly higher only in weeks 39 (Tukey *p* < 0.001) and 41 (Tukey *p* < 0.001).

### Principal component analysis of fatty acid percentage composition

Fifty-eight fatty acids were found in krill. Only fatty acids at percentages of ≥0.5% of total fatty acids (17 fatty acids) are analysed and presented in the following results.

Fatty acid percentage data for adult krill collected during weeks 1, 2, 4 and 5 (summer) were similar, and data collected during weeks 26–43 (winter and spring) were similar, so data were combined into these two separate groups for principal component analysis (PCA). Results of PCA analyses for individual weeks can be found in Supplementary Figs S1 and S2.

**Figure 2:**
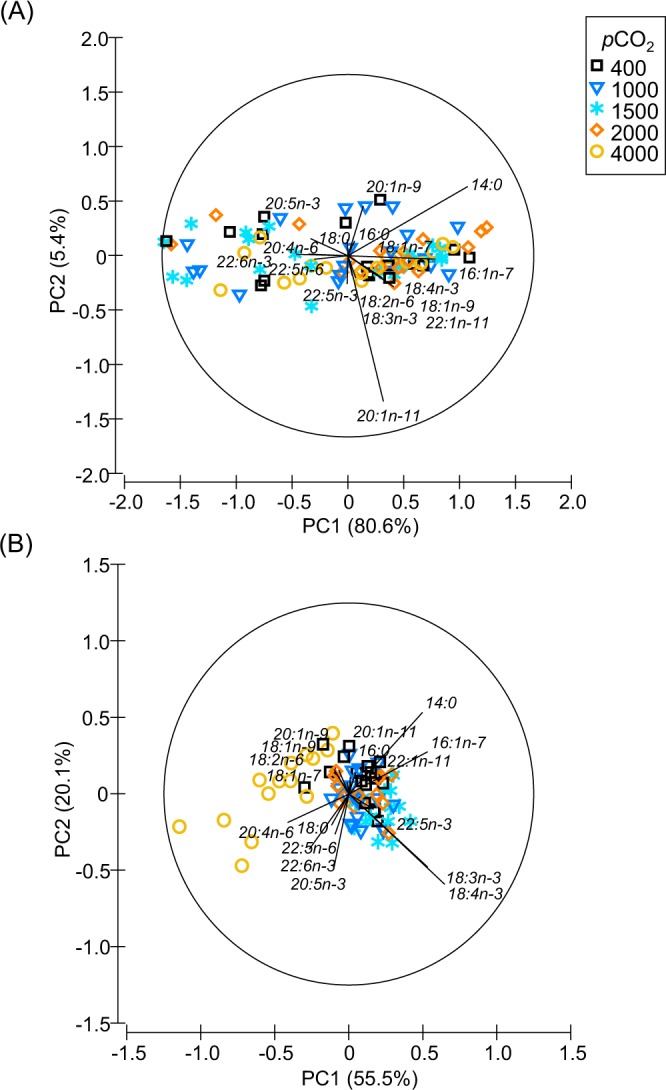
Principal component analyses of the fatty acid percentage composition of *Euphausia superba* in 400, 1000, 1500, 2000 and 4000 µatm *p*CO_2_ during **(A)** weeks 1–5 (summer) and **(B)** weeks 26–43 (winter and spring) of the one-year ocean acidification experiment. The amount of variation (%) explained by each principal component (PC) is shown on the x-axis (PC1) and y-axis (PC2).

Fatty acid percentage composition of krill did not differ between *p*CO_2_ treatments during weeks 1–5 when analysed using PCA (Fig. 2A). Principal component 1 (PC1) separated krill with higher percentages of LC-PUFA from those with higher percentages of 14:0 and medium-chain (C_16_–C_18_) monounsaturated fatty acids (MUFA) and PUFA, but no separation of *p*CO_2_ treatments was observed along PC1(x-axis) or principal component 2 (PC2; y-axis).

Krill fatty acid percentage composition differed between *p*CO_2_ treatments during weeks 26–43 (Fig. 2B). PC1 clearly separated krill in 4000 µatm *p*CO_2_ seawater from krill in other *p*CO_2_ treatments (Fig. 2B). Krill in 4000 µatm *p*CO_2_ seawater had higher percentages of LC-PUFA 20:4n-6 and 22:6n-3 than krill in 400–2000 µatm *p*CO_2_ seawater. Krill in 400–2000 µatm *p*CO_2_ treatments had higher percentages of 14:0, 16:1n-7, 18:3n-3 and 18:4n-3 (Fig. 2B). PC2 separated krill with higher percentages of 14:0 and 16:1n-7 (predominantly krill in 400 µatm *p*CO_2_) from those with higher percentages of 18:3n-3 and 18:4n-3 (predominantly krill in 1500 µatm *p*CO_2_) and those with higher percentages of 18:2n6, 18:1n9 and 20:5n-3 and 22:6n-3 (predominantly krill in 4000 µatm *p*CO_2_). Principal component loadings for each fatty acid included in the PCA analyses are shown in Supplementary Table S1.

Percentages (mean ± SD) of the eight fatty acids with the highest PCA loadings (14:0, 16:1n-7, 18:3n-3, 18:4n-3, 18:2n-6, 20:4n-6, 20:5n-3, 22:6n-3) in each *p*CO_2_ treatment are shown in Supplementary Table S2 (weeks 1–5; no significant differences, *p* > 0.05) and Table 1 (weeks 26–43).

**Table 1:** Percentage composition (mean ± SD) of selected fatty acids in *Euphausia superba* reared in 400, 1000, 1500, 2000 and 4000 *p*CO_2_ seawater during experimental weeks 26, 39, 41 and 43 of the one-year ocean acidification experiment.

Fatty Acid	*p*CO_2_	Week 26	Week 39	Week 41	Week 43
14:00	400	4.55 ± 0.59	4.96 ± 0.34	5.57 ± 0.51	5.10 ± 0.37
	1000	4.54 ± 0.40	4.92 ± 0.70	4.38 ± 0.43	4.88 ± 0.44
	1500	4.50 ± 0.76	5.05 ± 0.31	4.08 ± 1.13	4.90 ± 0.26
	2000	4.53 ± 0.28	4.92 ± 0.45	4.63 ± 0.68	4.85 ± 0.37
	4000	**2.98** ± **0.97** ↓	4.97 ± 0.38	**3.47** ± **0.15** ↓	4.17 ± 0.45
16:1n-7	400	5.55 ± 0.34	6.04 ± 0.27	6.50 ± 0.56	5.90 ± 0.47
	1000	5.42 ± 0.18	5.78 ± 0.56	5.66 ± 0.57	5.84 ± 0.38
	1500	5.83 ± 0.64	6.08 ± 0.43	5.83 ± 0.50	5.50 ± 0.53
	2000	5.30 ± 0.14	5.85 ± 0.65	5.77 ± 0.55	5.65 ± 0.44
	4000	**3.72** ± **1.06** ↓	**4.77** ± **0.55** ↓	**4.27** ± **0.06** ↓	**4.43** ± **0.47** ↓
18:2n-6	400	8.80 ± 0.36	9.10 ± 0.24	9.13 ± 0.78	9.56 ± 0.47
	1000	8.78 ± 0.29	9.12 ± 0.13	9.18 ± 0.25	9.30 ± 0.25
	1500	**8.28** ± **0.17** ↓	**8.70** ± **0.14** ↓	**8.85** ± **0.47** ↓	**8.93** ± **0.21** ↓
	2000	8.90 ± 0.18	9.30 ± 0.33	9.43 ± 0.59	9.18 ± 0.13
	4000	**8.90** ± **0.48** ↑	**10.07** ± **0.25** ↑	**10.30** ± **0.10** ↑	**9.80** ± **0.80** ↑
18:3n-3	400	3.10 ± 0.26	3.76 ± 0.50	3.67 ± 0.45	3.36 ± 0.51
	1000	3.80 ± 0.60	3.80 ± 0.50	3.84 ± 0.30	3.70 ± 0.20
	1500	**4.20** ± **0.22** ↑	**4.08** ± **0.26** ↑	**4.58** ± **0.36**↑	**4.10** ± **0.44** ↑
	2000	3.40 ± 0.18	3.77 ± 0.17	3.87 ± 0.25	4.10 ± 0.35
	4000	**2.68** ± **0.34** ↓	**2.67** ± **0.23** ↓	**2.40** ± **0.20** ↓	**2.67** ± **0.15** ↓
18:4n-3	400	0.62 ± 0.13	1.04 ± 0.27	0.93 ± 0.06	0.82 ± 0.19
	1000	0.84 ± 0.27	1.02 ± 0.29	1.08 ± 0.18	0.90 ± 0.14
	1500	**1.18** ± **0.22** ↑	**1.12** ± **0.15** ↑	**1.35** ± **0.25** ↑	**1.27** ± **0.35** ↑
	2000	0.72 ± 0.10	1.00 ± 0.14	0.93 ± 0.06	1.15 ± 0.26
	4000	**0.40** ± **0.25** ↓	**0.50** ± **0.00** ↓	**0.47** ± **0.06** ↓	**0.60** ± **0.10** ↓
20:4n-6	400	1.60 ± 0.24	1.52 ± 0.16	1.40 ± 0.10	1.42 ± 0.04
	1000	1.42 ± 0.18	1.52 ± 0.19	1.58 ± 0.13	1.50 ± 0.07
	1500	1.52 ± 0.10	1.32 ± 0.10	1.45 ± 0.17	1.30 ± 0.10
	2000	1.62 ± 0.10	1.40 ± 0.08	1.47 ± 0.21	1.38 ± 0.10
	4000	**2.38** ± **0.45** ↑	1.70 ± 0.10	**2.03** ± **0.31** ↑	1.63 ± 0.06
20:5n-3	400	6.08 ± 0.39	6.78 ± 0.80	6.90 ± 0.30	6.32 ± 0.56
	1000	6.20 ± 0.43	7.03 ± 0.79	7.54 ± 0.38	7.18 ± 0.49
	1500	7.12 ± 0.45	7.00 ± 0.22	7.62 ± 1.15	6.87 ± 0.25
	2000	6.58 ± 0.31	6.62 ± 0.42	6.70 ± 0.36	7.08 ± 0.33
	4000	**7.96** ± **1.15** ↑	6.53 ± 0.31	7.13 ± 0.47	7.07 ± 0.81
22:6n-3	400	10.62 ± 1.07	10.84 ± 0.36	9.70 ± 0.85	10.70 ± 0.54
	1000	10.42 ± 0.33	10.60 ± 0.47	11.18 ± 0.99	10.82 ± 0.41
	1500	10.57 ± 1.10	10.45 ± 0.37	10.78 ± 1.15	11.20 ± 0.72
	2000	10.55 ± 0.62	10.38 ± 1.13	10.70 ± 0.46	10.57 ± 0.41
	4000	**13.38** ± **2.38** ↑	10.23 ± 1.46	12.07 ± 0.76	10.70 ± 0.62

Values in bold type are those that are significantly different (Two way ANOVA *p*CO_2_*week and post-hoc comparisons; *p* < 0.05) to ambient seawater (400 µatm *p*CO_2_). Arrows illustrate whether there are lower levels (**↓**) or higher levels (**↑**) of the fatty acid in krill compared with those in ambient seawater. For each *p*CO_2_ treatment, n = 3–5.

### Fatty acid indicators of homeoviscous adaptation and immune responses in krill

**Figure 3:**
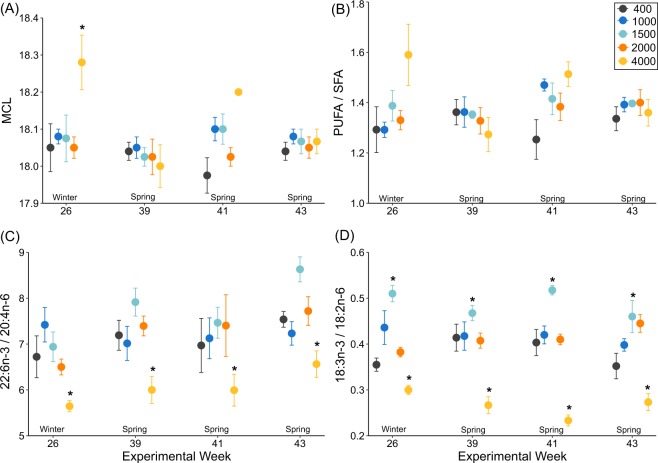
Fatty acid indicators of homeoviscous adaptation and immune responses in *Euphausia superba* exposed to 400, 1000, 1500, 2000 and 4000 µatm *p*CO_2_ in experimental weeks 26, 39, 41 and 43, where (**A**) Mean chain length (MCL); (**B**) ratio of polyunsaturated to saturated fatty acids (PUFA/SFA); (**C**) ratios of docosahexaenoic acid/arachidonic acid (22:6n-3/20:4n-6); (**D**) alpha-linolenic acid/linoleic acid (18:3n-3/18:2n-6). All values are mean ± SE. For each *p*CO_2_ treatment and week, n = 3–5. Statistically significant differences to ambient seawater (400 µatm *p*CO_2_) are highlighted with an asterisk (*p* < 0.05).

Seawater *p*CO_2_ did not affect the MCL, PUFA/SFA ratio, 22:6n-3/20:4n-6 ratio and 18:3n-3/18:2n-6 ratio in krill during weeks 1–5 (Supplementary Table S3), but these ratios were altered during weeks 26–43 (Fig. 3). MCL was higher in krill in 4000 µatm *p*CO_2_ than krill in other treatments in week 26 (Two Way ANOVA *p*CO_2_^*^week *p* = 0.049, Tukey *p* < 0.05). The PUFA/SFA ratio did not differ between krill in different *p*CO_2_ treatments (Two Way ANOVA *p*CO_2_^*^week, *p* = 0.089; *p*CO_2_, *p* = 0.101). Krill in 4000 µatm *p*CO_2_ had a lower 22:6n-3/20:4n-6 ratio than krill in other treatments during weeks 26, 39, 41 and 43 (Two Way ANOVA *p*CO_2_^*^week = 0.571; *p*CO_2_, Tukey *p* < 0.001). Krill in 1500 µatm *p*CO_2_ had higher 18:3n-3/18:2n-6 ratios than krill in other treatments (Two Way ANOVA *p*CO_2_*week *p* = 0.100; *p*CO_2_ Tukey *p* < 0.001) and krill in 4000 µatm *p*CO_2_ had lower 18:3n-3/18:2n-6 ratios than krill in other treatments during all weeks from 26–43 (ANOVA *p*CO_2_^*^week, *p* = 0.100; *p*CO_2,_ Tukey *p* < 0.05).

## Discussion

Krill reared in ambient seawater *p*CO_2_ levels (400 µatm *p*CO_2_), and those levels predicted for the near-future (100–300 years; 1000–2000 µatm *p*CO_2_) did not have significantly different quantities of total lipid (mg g^−1^ DM) or ratios of PL/ST. We observed no effects of near-future *p*CO_2_ on fatty acid composition during weeks 1–5 of the experiment (summer). In winter and spring (weeks 26–43), elevated percentages of C_18_ fatty acids 18:4n-3 and 18:3n-3 were measured in krill in 400–2000 µatm *p*CO_2_ and were highest in krill in 1500 µatm *p*CO_2_. Krill in extreme *p*CO_2_ (4000 µatm), had a different lipid composition to those in 400–2000 µatm *p*CO_2_ treatments during winter and spring. Some krill had lower quantities of total lipid, higher PL/ST ratios and MCL, and all krill had consistently lower ratios of n-3/n-6 fatty acids (22:6n-3/20:4n-6 and 18:3n-3/18:2n-6).

The absence of any *p*CO_2_ effect on krill biochemistry during the first five weeks of the experiment suggests that it may take some time before changes can be detected in the lipid profile of adult krill. Numerous short-term ocean acidification studies have, however, detected changes in the lipid and fatty acid profile of other organisms, over time periods substantially shorter than or equal to five weeks^23,27,30,34,35^. Krill metabolism is controlled by endogeneous rhythms which are cued by seasonal changes in photoperiod, and krill have higher metabolic rates during summer^42^. A higher metabolic rate may enable krill to more efficiently regulate acid-base balance and other vital functions such as their lipid biochemistry. This could explain why effects of extreme *p*CO_2_ on krill biochemistry were not observed during summer, but were most evident in winter (week 26) when metabolic rates are lowest. The interaction between seasonal metabolic rates, lipid biochemistry and increased *p*CO_2_ at different time scales is a topic for further study.

Many ocean acidification studies to date have found no effect of near-future *p*CO_2_ on total lipid levels in organisms^11,31,33,34,43–45^. Lipids are an important energy source and essential for physiological function and survival, therefore, organisms are likely to maintain relative lipid levels unless they are under substantial physiological stress.

Like other laboratory studies^46,47^, krill in our study displayed seasonal fluctuations in lipid mass even when given a constant food supply. This occurred in all *p*CO_2_ treatments, indicating that endogenous rhythms entrained by the seasonal light cycle were the dominant driver controlling lipid deposition in krill^47^.

Our finding that near-future *p*CO_2_ did not have a significant effect on total lipid mass in adult krill suggests that ocean acidification does not affect their ability to feed or store fat. This corresponds well with a recent study^41^, which indicates that physiological processes in adult krill are unaffected by near-future acidification. Animals may preferentially retain lipids and utilize protein as an energy source when exposed to near-future *p*CO_2_^43^, or maintain lipid and protein levels but grow at a slower rate^33^. Adult krill in near-future *p*CO_2_, however, do not display slow or delayed growth compared with those in ambient seawater^41^. A previous study found that krill exposed to 750 µatm *p*CO_2_ for 24 hours had slightly lower protein content than krill in ambient *p*CO_2_ seawater^48^, suggesting that they may switch from lipid to protein catabolism in high *p*CO_2_ conditions.

Near-future *p*CO_2_ did not significantly alter the composition of fatty acids associated with immune function (n-3/n-6 ratios) and cell membrane fluidity (MCL, PUFA/SFA and PL/ST) in krill. This is a further indication that these levels of *p*CO_2_ do not induce physiological stress. Cell membrane fatty acid composition is tightly regulated by temperature^49^ and may be driven more by the cold temperatures krill are adapted to^14^. Ambient seawater temperatures (0.5 °C) were used in this study, which could explain the stability of these fatty acid ratios. Elevated seawater temperatures may influence fatty acid composition more than acidification, although previous studies indicate that krill lipids are not altered by temperatures up to 4 °C above ambient^46^.

Decreases in total lipid and increases in levels of inflammatory n-6 PUFA in krill reared in 4000 µatm *p*CO_2_, suggest that unlike krill in 400–2000 µatm *p*CO_2_, these krill were physiologically stressed. Lipid depletion observed during selected weeks in winter and spring corresponds with decreases in quantities of storage lipid (triacylglycerol) in these krill^41^. Physiological processes such as growth and maturation, along with acid-base regulation required in extreme seawater *p*CO_2_, are energetically expensive^50^ and these processes may have depleted lipid reserves. Feeding in these krill may have also been compromised and caused a decrease in total lipid, although feeding rates were not measured in this study.

Krill in 4000 µatm *p*CO_2_ seawater may have been storing 20:4n-6 for production of inflammatory eicosanoids, and for ion transport^15,51^, in an attempt to regulate immune responses and maintain intra- and extra-cellular pH in this extreme environment. Such increases in n-6 fatty acids have been observed in fish^28^ and shrimp^30^ exposed to acidification. Inflammation is important for organism health and tissue repair, but excessive inflammation is maladaptive^52^.

As levels of n-6 fatty acids in organisms increase, levels of n-3 fatty acids decrease, as the elongation-desaturation pathways for n-3 and n-6 fatty acids compete for the same enzymes^53^. The lower n-3/n-6 ratios in krill in 4000 µatm *p*CO_2_ during winter and spring may, therefore, correspond to a shift in elongation-desaturation pathways used by these krill. The increase in n-3 PUFA in krill up to 1500 µatm *p*CO_2_, followed by a decrease down to 4000 µatm *p*CO_2_, suggests that 1500 µatm *p*CO_2_ may be the point at which krill fatty acid composition switches from an anti-inflammatory status (more n-3 PUFA) to a pro-inflammatory status (more n-6 PUFA).

Cell membrane alteration via homeoviscous adaptation has been most commonly explored with respect to changing temperatures^10,54^, but other factors such as salinity, hypoxia^18^, and pH^11,21^ can alter membrane structure. The higher ratios of PL/ST in krill in 4000 µatm *p*CO_2_ in winter and spring suggests that krill may have been actively increasing membrane fluidity, to enable more efficient exchange of ions across their cell membranes and control acid-base balance. Alternatively, the ability of krill in 4000 µatm *p*CO_2_ to maintain an optimal ST composition may have been compromised. This could lead to membrane ‘hyper-fluidity’ and disrupt cellular function^11^. Under hypercapnic stress, homeoviscous adaptation through regulation of lipid class ratios (e.g. PL/ST) may be more energy efficient than modification of PUFA/SFA ratios and MCL, which remained more stable in krill in 4000 µatm *p*CO_2_.

The fatty acid profile of krill in our laboratory study also reflects their aquarium diet and does differ to that of wild krill. Ratios of 22:6n-3/20:4n-6, 20:5n-3/22:6n-3, and 18:3n-3/18:2n-6 are higher in wild krill^3,36^ than were observed for our laboratory reared krill, indicating that wild krill have higher n-3/n-6 ratios. The diet of wild krill is not replicable in the laboratory^55^, but the higher n-3/n-6 ratios of these krill may influence and even further enhance their resilience to elevated *p*CO_2_, due to their higher levels of anti-inflammatory fatty acids. Levels of krill prey in the Southern Ocean also fluctuate spatially and temporally^37^, and krill in our study were fed a constant supply of food. Krill increase their feeding rates when exposed to high *p*CO_2_^48^, possibly to maintain enough energy for physiological processes under *p*CO_2_ stress^41,48^. Changing food levels both seasonally, regionally and with climate change may, therefore, also influence how wild krill respond to ocean acidification.

Krill will be exposed to multiple climate change stressors in the future, in addition to ocean acidification^4^. Rapid warming is already evident in the West Antarctic Peninsula region^56^, both at the sea surface^57^, and in the deep ocean (Antarctic Bottom Water^58^). In laboratory studies, simulated ocean warming significantly affects the fatty acid composition of some organisms^11,26,29,59^. A previous long-term laboratory study found only minor differences between the lipid and fatty acid composition of krill reared in −1 °C, 1 °C and 3 °C^46^. The temperature range used in this study was within the range that krill experience in their natural environment (krill are abundant at South Georgia where seawater temperatures reach 5 °C^37^), therefore, the temperatures may not have been high enough to detect significant temperature effects. Further studies are needed to establish whether the combined effects of increased seawater temperature and *p*CO_2_ affect the lipid and fatty acid composition of krill.

## Conclusions

Lipid mass and fatty acid composition in adult krill were unaffected when krill were exposed to near-future levels of *p*CO_2_ (1000–2000 µatm) in the laboratory. Extreme *p*CO_2_ altered the lipid and fatty acid content and composition of krill, although consistent differences were not observed across all experimental weeks. Extreme *p*CO_2_ had no effect on krill lipid biochemistry during summer, but during selected weeks in winter and spring, krill in 4000 µatm *p*CO_2_ had elevated levels of inflammatory omega-6 fatty acids and showed evidence of increased membrane fluidity. These observations suggest that krill may be less able to tolerate elevated *p*CO_2_ conditions during winter and spring, when metabolic rates are lower and reproductive maturation occurs. Seawater pH levels are also lower in the Antarctic in the winter than summer^60^, and prey availability is lower in winter in some areas of the Southern Ocean^37^. Collectively, these factors may influence how krill respond to near-future *p*CO_2_ in the wild, and determine their resilience in a future high CO_2_ world.

## Methods

### Experimental conditions

Experimental conditions are described in detail in a previous manuscript^41^. Briefly, krill were collected from the Southern Ocean (66–03°S, 59–25°E and 66–33°S, 59–35°E) on the research and supply vessel (RSV) *Aurora Australis*, using a mid-water trawl net (sampled within the top 100 m of the water column). They were held in shipboard aquaria using standard husbandry methods^61^, and transported to the Australian Antarctic Division Krill Aquarium in Tasmania.

For ocean acidification experiments, five 300 L tanks were equilibrated to five *p*CO_2_ levels; 400 μatm *p*CO_2_ (pH 8.1 control treatment), 1000 μatm *p*CO_2_ (pH 7.8), 1500 μatm *p*CO_2_ (pH 7.6), 2000 μatm *p*CO_2_ (pH 7.4) and 4000 μatm *p*CO_2_ (pH 7.1). Seawater temperature of all tanks was held at 0.5 °C (±0.2). Seawater chemistry for the duration of the experiment is reported in the supplementary material of a previous manuscript^41^. Observational units (CO_2_ treatment tanks) could not be replicated, due to the large tank size required to achieve the best possible animal husbandry for this pelagic species, and the limited space and resources available for these large tanks over such a long-term study. Tanks were inspected daily, and there was no visual evidence to suggest that tank effects were confounding our experimental results.

Two hundred krill were randomly assigned to each tank on the first day of the experiment (25^th^ January 2016), and reared in these *p*CO_2_ treatments until the experiment ended on the 12^th^ December 2016. Light was controlled in the laboratory to mimic the seasonal Southern Ocean light regime (66°S, 30 m depth) and krill were fed six days per week with a mixed microalgal diet of the Antarctic species *Pyramimonas gelidicola* (2 × 10^4^ cells mL^−1^), and Reed Mariculture Inc. (USA) cultures of *Thalassiosira weissflogii* (8.8 × 10^3^ cells mL^−1^), *Pavlova lutheri* (4.5 × 10^4^ cells mL^−1^) and *Isochryisis galbana* (5.5 × 10 cells mL^−1^).

### Sample collection and lipid extraction

Krill were sampled from the *p*CO_2_ treatment tanks in experimental weeks 1, 2, 4 and 5 (summer), 26 (winter), and 39, 41 and 43 (spring). Five to ten krill were sampled from each tank during each sampling week (only three krill were sampled from the 4000 μatm *p*CO_2_ tank due to increased mortality in that tank and lower overall numbers of krill^41^). Individual krill were placed in cryo-tubes and frozen immediately at −80 °C until needed for lipid analysis.

Krill were weighed (wet mass), and the length of each specimen was measured from the tip of the rostrum to the tip of the uropod using measurement ‘Standard Length 1’^62^. To prevent sample degradation, krill were kept frozen during the measuring process. A dry mass (g) for each krill sample was obtained by multiplying the wet mass by 0.2278 to account for the 77.2% water content in krill^2^.

Krill specimens were added to separatory funnels and extracted using a modified Bligh and Dyer method^63^, consisting of a methanol:dichloromethane:water (MeOH:CH_2_Cl_2_:H_2_O) solvent mixture (20:10:7 mL), and overnight extraction. Phase separation was carried out the following day by adding 10 mL CH_2_Cl_2_ and 10 mL saline MilliQ H_2_O to each separatory funnel, giving a final MeOH:CH_2_Cl_2_:H_2_O solvent ratio of 1:1:0.85. The lower layer was drained into a round bottomed flask, and the total solvent extract was concentrated using rotary evaporation. The concentrated extract was transferred into a pre-weighed 2 mL vial and the solvent was blown down under nitrogen (N_2_) gas to obtain a total lipid extract (TLE) weight. Solvent (CH_2_Cl_2_) was added until further procedures were carried out to avoid oxidation.

### Lipid class analysis

TLE were used to obtain the lipid class composition of each sample. Aliquots (1 μl) of each TLE were spotted on chromarods and developed in a solvent bath of hexane:diethyl-ether:acetic acid (90:10:0.1 mL, v-v:v) for 25 min, before drying in an oven at 50 °C for 10 min. Chromarods were placed in an Iatroscan MK-5 TLC/FID analyser (Iatron Laboratories, Tokyo, Japan) for analysis. A standard solution of known quantities of wax esters (WE), triacylglycerols (TAG), free fatty acids (FFA), sterols (ST), and phospholipids (PL) was used to confirm peak identities and to calibrate the flame ionisation detector. Lipid class peaks were labelled using SIC-480II Iatroscan Integrating Software v.7.0-E, quantified using predetermined linear regressions, and expressed as mg per g of krill dry mass (mg g DM^−1^). Triacylglycerol data is presented in an earlier manuscript^41^. Only the PL to ST ratio is presented in this manuscript as we were primarily interested in investigating homeoviscous adaptation in krill.

### Fatty acid analysis

To prepare fatty acid methyl esters (FAME), a subsample of the TLE was transferred to a glass test tube fitted with a Teflon lined screw cap, and treated with 3 mL methylating solution (MeOH: CH_2_Cl_2_: HCl (hydrochloric acid), 10:1:1, v-v:v). The sample was then heated at 90–100 °C for 1 hr 15 mins. Samples were cooled and 1 mL of H_2_O and 1.8 mL of C_6_H_14_ (hexane): CH_2_Cl_2_ solution was added to extract the FAME. Samples were then centrifuged for five minutes at 3000 rpm, and the upper layer containing FAME was transferred to a vial. An additional 1.8 mL of C_6_H_14_:CH_2_Cl_2_ was added to the test tube and samples were centrifuged again. This process was repeated three times in total, and samples were blown down using N_2_ gas in between transfers. FAME samples were made up to 1.5 mL with CH_2_Cl_2_ and stored at −20 °C until further analysis. Prior to analysis, samples were blown down again using N_2_ gas and 1.5 mL of internal injection standard (23:0 FAME) was added to each vial.

Samples were analysed via gas chromatography (GC-FID) using an Agilent Technologies 7890 A GC System (Palo Alto, California USA) equipped with a non-polar Equity™−1 fused silica capillary column (15 m × 0.1 mm internal diameter and 0.1 µm film thickness). Samples (0.2 µl) were injected in splitless mode at an oven temperature of 120 °C with helium as the carrier gas. The oven temperature was raised to 270 °C at a rate of 10 °C per minute, then to 310 °C at 5 °C per minute. Agilent Technologies ChemStation software was used to quantify fatty acid peaks, with initial identification based on comparison of retention times with known (Nu Chek Prep mix) and laboratory (fully characterised tuna oil) standards. Fatty acid peaks were expressed as a percentage of the total fatty acid area. Fatty acid quantities (in mg g^−1^ DM and mg g^−1^ lipid) were calculated using the internal injection standard (C23:0) of known concentration.

Confirmation of component identification was performed by gas chromatography-mass spectrometry (GC-MS) of selected samples and was carried out on a ThermoScientific 1310 GC coupled with a TSQ triple quadruple. Samples were injected using a Tripleplus RSH auto sampler using a non polar HP-5 Ultra 2 bonded-phase column (50 m × 0.32 mm i.d. × 0.17 µm film thickness). The HP-5 column was of similar polarity to the column used for GC analyses. The initial oven temperature of 45 °C was held for 1 min, followed by an increase in temperature of 30 °C per minute to 140 °C, then at 3 °C per minute to 310 °C, where it was held for 12 minutes. Helium (He) was used as the carrier gas. The operating conditions of the GC-MS were: electron impact energy 70 eV; emission current 250 µamp, transfer line 310 °C; source temperature 240 °C; scan rate 0.8 scan/sec and mass range 40–650 Da. Thermo Scientific Xcalibur^TM^ software (Waltham, MA, USA) was used to process and acquire mass spectra.

Mean fatty acid chain length (MCL) was calculated using the equation from reference^11^:

$$\begin{array}{rcl}{\rm{MCL}} & = & \sum ({\rm{mg}}\,{\rm{fatty}}\,{\rm{acid}}\,{{\rm{g}}}^{-1}\,{\rm{lipid}}\times {\rm{C}})/{\rm{total}}\,{\rm{mg}}\,{\rm{fatty}}\,{\rm{acid}}\,{{\rm{g}}}^{-1}\,{\rm{lipid}}\\  &  & \,\,\,\,where\,{\rm{C}}={\rm{number}}\,{\rm{of}}\,{\rm{carbon}}\,{\rm{atoms}}\end{array}$$

### Statistical analyses

Principal component analyses (PCA) were carried out in PRIMER 6 (http://www.primer-e.com). Pearson correlation was used due to differences in fatty acid variances, and data were transformed (log x + 1) before analysis. All other statistical analyses were carried out in RStudio (v 1.1.453; www.rstudio.com). Total lipid, specific fatty acids, lipid class and fatty acid ratios were analysed using Two Way ANOVA with *p*CO_2_ and week as main effects, and a *p*CO_2_*week interaction term. Tukey comparisons or Dunnett’s tests were used to compare levels of *p*CO_2_ with one another. On visual assessment of the data, weeks 1–5 were analysed as a group, and weeks 26–43 were analysed as a separate group, as the groups had heterogeneous variances and represented two distinct data sets. Type 3 Sums of Squares were used as the sampling regime was unbalanced. Data were visualised using Q-Q plots and residuals versus fitted values plots, to verify that they met the assumptions of normality and homogeneity of variances. Log or square root transformations were applied when assumptions of normality and/or homogeneity of variances were not met. For Two Way ANOVA of total lipid data, one outlier was removed from the statistical analysis in order to meet assumptions of homogeneity of variances. All tests were two tailed with α = 0.05.

Principal component figures were created in PRIMER 6, and all other figures were created using the RStudio packages ggplot2, plyr and dplyr.

## Supplementary information

Supplementary Material

## Data Availability

The datasets generated during and/or analysed during the current study are available from the corresponding author on reasonable request.
