# Supplementary material for: Near-future ocean acidification does not alter the lipid content and fatty acid composition of adult Antarctic krill
Source: Sci Rep. 2019 Aug 26;9:12375. doi: 10.1038/s41598-019-48665-5 (PMC6710253; doi:10.1038/s41598-019-48665-5)
Supplement: Supplementary file 1 — Supplementary Material [file 41598_2019_48665_MOESM1_ESM.docx]

## Supplementary Information

#

# Near-future ocean acidification does not alter the lipid content and fatty acid composition of adult Antarctic krill

Jessica. A. Ericson^1,2,3,4*^, Nicole Hellessey^1,2,3^, So Kawaguchi^2,5^, Peter D. Nichols^1,3^, Stephen Nicol^1^, Nils Hoem^6^, Patti Virtue^1,2,3^

^1^Institute for Marine and Antarctic Studies, University of Tasmania, 20 Castray Esplanade, Battery Point, Tasmania 7004, Australia

^2^Antarctic Climate & Ecosystems Cooperative Research Centre, 20 Castray Esplanade, Battery Point, Tasmania 7004, Australia

^3^CSIRO Oceans and Atmosphere, Castray Esplanade, Battery Point, Tasmania 7004, Australia

^4^ Cawthron Institute, 98 Halifax Street East, The Wood, Nelson 7010, New Zealand

^5^Australian Antarctic Division, 203 Channel Highway, Kingston, Tasmania 7050, Australia

^6^Aker Biomarine, Oksenøyveien 10, P.O. Box 496, NO-1327, Lysaker, Norway

*Corresponding author: jessica.ericson@utas.edu.au

**Figure S1.** Principal component analyses for the fatty acid composition (%) of *Euphausia superba* in separate weeks 1, 2, 4 and 5 of the one-year ocean acidification experiment

**Figure S2.** Principal component analyses for the fatty acid composition (%) of *Euphausia superba* in separate weeks 26, 39, 41 and 43 of the one-year ocean acidification experiment

| **Table S1.** Percentage variation and cumulative variation, and loadings for principal component analyses of *Euphausia superba* fatty acid composition (%) in combined weeks 1 – 5 and weeks 26 – 43 of the ocean acidification experiment. | | | | |
| --- | --- | --- | --- | --- |
|  | **Weeks 1 – 5** | | **Weeks 26 – 43** | |
| **Principal Component** | **PC1** | **PC2** | **PC1** | **PC2** |
| **Variation (%)** | 80.6 | 5.4 | 55.5 | 20.1 |
| **Cumulative Variation (%)** | **-** | 86.0 | - | 75.6 |
|  |  |  |  |  |
| **Loadings** |  |  |  |  |
| 14:0 | 0.644 | 0.381 | 0.398 | 0.428 |
| 16:0 | 0.036 | 0.081 | 0.007 | 0.090 |
| 16:1n-7c | 0.471 | -0.019 | 0.426 | 0.222 |
| 18:0 | -0.003 | 0.090 | -0.094 | -0.162 |
| 18:1n-7c | 0.007 | -0.027 | -0.008 | 0.062 |
| 18:1n-9c | 0.185 | -0.123 | -0.079 | 0.128 |
| 18:2n-6 | 0.023 | -0.110 | -0.055 | 0.126 |
| 18:3n-3 | 0.042 | -0.123 | 0.426 | -0.382 |
| 18:4n-3 | 0.159 | -0.104 | 0.518 | -0.472 |
| 20:1n-9c | 0.083 | 0.275 | -0.069 | 0.109 |
| 20:1n-11 | 0.191 | -0.804 | 0.037 | 0.114 |
| 20:4n-6 | -0.213 | 0.009 | -0.332 | -0.149 |
| 20:5n-3 | -0.201 | 0.094 | -0.080 | -0.379 |
| 22:1n-11 | 0.246 | -0.171 | 0.012 | 0.060 |
| 22:5n-3 | -0.004 | -0.157 | 0.134 | -0.080 |
| 22:5n-6 | -0.181 | -0.020 | -0.075 | -0.211 |
| 22:6n-3 | -0.279 | 0.008 | -0.214 | -0.289 |

| **Table S2.** Percentage composition (mean ± SD) of selected fatty acids in *Euphausia superba* reared in 400, 1000, 1500, 2000 and 4000 *p*CO_2_ seawater in experimental weeks 1, 2, 4 and 5 of the one-year ocean acidification experiment. No significant differences were found between the control treatment (400 µatm *p*CO_2_) and any of the elevated *p*CO_2_ treatments (1000 – 4000 µatm *p*CO_2_ ) during weeks 1, 2, 4 or 5 (*p* > 0.05). For each *p*CO_2_ treatment n = 3 – 7. | | | | | |
| --- | --- | --- | --- | --- | --- |
| Fatty Acid | *p*CO_2_ | Week 1 | Week 2 | Week 4 | Week 5 |
| 14:0 | 400  1000  1500  2000  4000 | 1.35 ± 0.61  1.30 ± 1.35  0.90 ± 1.42  1.50 ± 1.33  1.45 ± 0.86 | 1.07 ± 0.72  2.10 ± 1.91  1.02 ± 0.87  3.72 ± 1.86  1.25 ± 0.91 | 2.52 ± 0.44  2.85 ± 0.56  1.80 ± 1.13  2.12 ± 1.18  2.00 ± 1.12 | 2.48 ± 2.02  2.38 ± 1.21  2.83 ± 1.49  3.26 ± 1.15  3.52 ± 0.94 |
| 16:1n-7c | 400  1000  1500  2000  4000 | 2.73 ± 0.96  2.75 ± 1.80  1.98 ± 1.18  2.65 ± 1.44  3.05 ± 0.89 | 2.65 ± 1.07  3.98 ± 1.74  2.46 ± 1.01  5.20 ± 1.22  2.52 ± 1.02 | 4.30 ± 0.36  4.05 ± 1.14  3.48 ± 1.52  3.30 ± 1.54  3.08 ± 1.46 | 3.86 ± 2.21  3.16 ± 0.86  4.17 ± 1.86  4.54 ± 0.91  4.65 ± 0.69 |
| 18:2n-6 | 400  1000  1500  2000  4000 | 7.30 ± 0.52  7.25 ± 0.82  7.45 ± 0.31  7.05 ± 0.37  7.42 ± 0.66 | 7.38 ± 0.46  7.15 ± 0.59  7.22 ± 0.93  7.30 ± 0.75  7.12 ± 0.38 | 8.00 ± 0.32  7.05 ± 0.69  7.56 ± 0.29  7.32 ± 0.89  7.44 ± 0.69 | 7.38 ± 0.63  7.40 ± 0.37  7.40 ± 0.41  7.58 ± 0.42  7.47 ± 0.46 |
| 18:3n-3 | 400  1000  1500  2000  4000 | 2.38 ± 0.26  2.32 ± 0.26  2.27 ± 0.32  2.33 ± 0.13  2.27 ± 0.17 | 2.42 ± 0.42  2.42 ± 0.30  2.36 ± 0.59  2.30 ± 0.32  2.32 ± 0.05 | 2.65 ± 0.17  2.48 ± 0.59  2.44 ± 0.23  2.34 ± 0.39  2.30 ± 0.52 | 2.58 ± 0.40  2.66 ± 0.11  2.40 ± 0.29  2.66 ± 0.38  2.60 ± 0.39 |
| 18:4n-3 | 400  1000  1500  2000  4000 | 0.18 ± 0.21  0.17 ± 0.23  0.12 ± 0.25  0.25 ± 0.21  0.25 ± 0.21 | 0.22 ± 0.26  0.35 ± 0.24  0.28 ± 0.22  0.42 ± 0.08  0.30 ± 0.14 | 0.40 ± 0.08  0.42 ± 0.13  0.26 ± 0.17  0.28 ± 0.18  0.30 ± 0.19 | 0.32 ± 0.22  0.28 ± 0.08  0.39 ± 0.20  0.46 ± 0.18  0.40 ± 0.08 |
| 20:4n-6 | 400  1000  1500  2000  4000 | 2.62 ± 0.36  2.67 ± 0.64  3.17 ± 0.68  2.80 ± 0.42  2.73 ± 0.56 | 2.83 ± 0.51  2.15 ± 0.79  2.94 ± 0.56  1.90 ± 0.43  2.95 ± 0.74 | 2.17 ± 0.17  2.45 ± 0.54  2.68 ± 0.79  2.62 ± 0.64  2.62 ± 0.49 | 2.72 ± 1.14  2.74 ± 0.45  2.47 ± 0.87  2.16 ± 0.30  2.18 ± 0.38 |
| 20:5n-3 | 400  1000  1500  2000  4000 | 13.90 ± 2.59  13.93 ± 3.85  13.97 ± 2.28  13.75 ± 2.20  12.53 ± 1.10 | 13.15 ± 1.74  11.68 ± 1.93  14.50 ± 3.08  10.44 ± 0.26  14.40 ± 1.79 | 10.65 ± 1.36  12.18 ± 1.23  12.44 ± 1.86  12.70 ± 2.68  12.06 ± 2.70 | 12.40 ± 3.32  13.06 ± 1.63  12.20 ± 2.39  11.00 ± 0.91  10.55 ± 0.66 |
| 22:6n-3 | 400  1000  1500  2000  4000 | 19.07 ± 2.74  20.33 ± 5.87  20.80 ± 4.85  20.23 ± 4.57  17.88 ± 2.42 | 20.95 ± 3.96  15.90 ± 3.94  21.26 ± 4.51  14.16 ± 2.34  20.82 ± 4.36 | 14.82 ± 0.62  16.43 ± 1.89  18.28 ± 4.10  18.22 ± 4.38  17.58 ± 4.92 | 18.74 ± 6.66  19.40 ± 2.67  17.24 ± 5.61  15.04 ± 1.56  14.05 ± 1.81 |

| **Table S3.** Mean chain length (MCL), ratios of polyunsaturated to saturated fatty acids (PUFA/SFA), ratios of 22:6n-3/20:4n-6 (DHA/ARA), and ratios of 18:3n-3/18:2n-6 (ALA/LA) in *Euphausia superba* sampled in experimental week’s 1 – 5 (Jan – Feb; summer) of the one-year ocean acidification experiment. No significant differences were found between the control treatment (400 µatm *p*CO_2_) and any of the elevated *p*CO_2_ treatments (1000 – 4000 µatm *p*CO_2_ ) during weeks 1, 2, 4 or 5 (*p* > 0.05). Units are mean ± SD. For each  *p*CO_2_ treatment n = 3 – 7. | | | | | |
| --- | --- | --- | --- | --- | --- |
|  | *p*CO_2_ | MCL | PUFA/SFA | DHA/ARA | ALA/LA |
| Week 1 | 400 | 18.75 ± 0.17 | 2.27 ± 0.26 | 7.31 ± 0.92 | 0.32 ± 0.04 |
|  | 1000 | 18.77 ± 0.15 | 2.41 ± 0.66 | 7.57 ± 0.56 | 0.32 ± 0.02 |
|  | 1500 | 18.82 ± 0.30 | 2.48 ± 0.58 | 6.56 ± 0.78 | 0.30 ± 0.03 |
|  | 2000 | 18.77 ± 0.31 | 2.40 ± 0.47 | 7.20 ± 0.86 | 0.33 ± 0.01 |
|  | 4000 | 18.68 ± 0.17 | 2.15 ± 0.29 | 6.63 ± 0.54 | 0.30 ± 0.01 |
|  |  |  |  |  |  |
| Week 2 | 400 | 18.85 ± 0.24 | 2.69 ± 0.54 | 7.41 ± 0.23 | 0.33 ± 0.04 |
|  | 1000 | 18.50 ± 0.37 | 1.96 ± 0.61 | 7.63 ± 0.98 | 0.34 ± 0.02 |
|  | 1500 | 18.88 ± 0.29 | 2.71 ± 0.55 | 7.23 ± 0.60 | 0.32 ± 0.05 |
|  | 2000 | 18.32 ± 0.22 | 1.67 ± 0.35 | 7.56 ± 0.68 | 0.32 ± 0.03 |
|  | 4000 | 18.85 ± 0.31 | 2.80 ± 0.84 | 7.17 ± 0.86 | 0.32 ± 0.02 |
|  |  |  |  |  |  |
| Week 4 | 400 | 18.45 ± 0.06 | 1.88 ± 0.12 | 6.86 ± 0.71 | 0.33 ± 0.03 |
|  | 1000 | 18.52 ± 0.15 | 1.90 ± 0.26 | 6.84 ± 0.92 | 0.35 ± 0.06 |
|  | 1500 | 18.64 ± 0.24 | 2.24 ± 0.48 | 6.94 ± 0.65 | 0.32 ± 0.03 |
|  | 2000 | 18.60 ± 0.33 | 2.11 ± 0.46 | 6.96 ± 0.35 | 0.32 ± 0.03 |
|  | 4000 | 18.56 ± 0.34 | 2.01 ± 0.55 | 6.63 ± 0.67 | 0.31 ± 0.04 |
|  |  |  |  |  |  |
| Week 5 | 400 | 18.60 ± 0.44 | 2.26 ± 0.87 | 7.12 ± 0.94 | 0.35 ± 0.01 |
|  | 1000 | 18.72 ± 0.19 | 2.37 ± 0.39 | 7.11 ± 0.43 | 0.36 ± 0.01 |
|  | 1500 | 18.54 ± 0.37 | 2.06 ± 0.63 | 7.03 ± 0.39 | 0.32 ± 0.03 |
|  | 2000 | 18.38 ± 0.11 | 1.78 ± 0.15 | 7.02 ± 0.75 | 0.35 ± 0.05 |
|  | 4000 | 18.30 ± 0.14 | 1.64 ± 0.24 | 6.50 ± 0.37 | 0.34 ± 0.03 |
